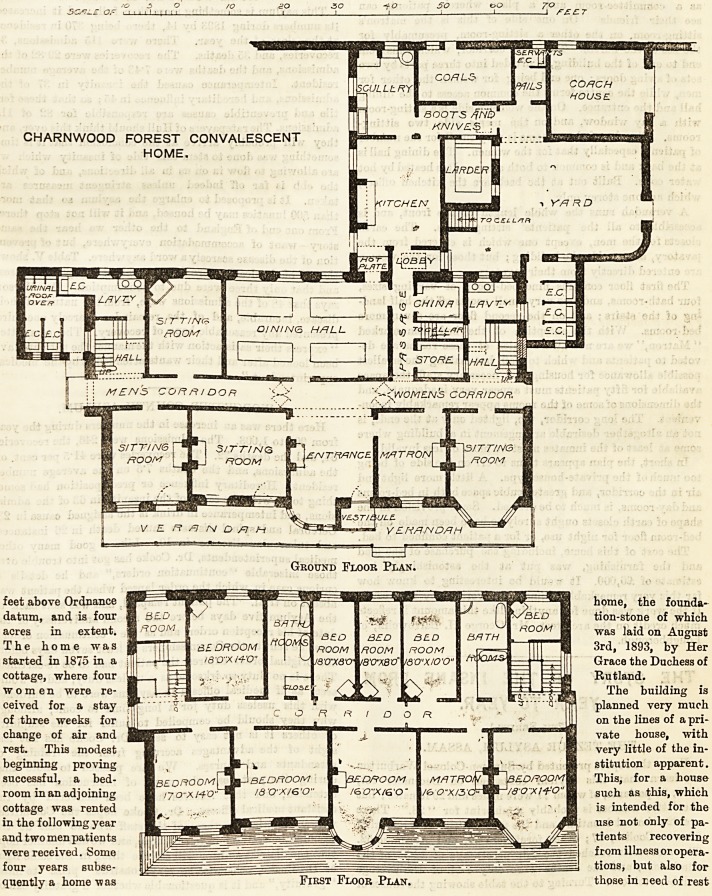# Hospital Construction

**Published:** 1894-10-06

**Authors:** 


					Oct. 6, 1894. THE HOSPITAL. 13
The Institutional Workshop.
HOSPITAL CONSTRUCTION.
CHARNWOOD FOREST CONVALESCENT HOME.
This institution is situated in the heart of Charnwood
Forest, near Woodhouse, and about three and a half
miles from Loughborough Station. This site is an
elevated one, beiug about four hundred and fifty
feet above Ordnance
datum, and is four
acres in extent.
The home was
started in 1875 in a
cottage, where four
women were re-
ceived for a stay
of three weeks for
change of air and
rest. This modest
beginning proving
successful, a bed-
room in an adjoining
cottage was rented
in the following year
and two men patients
were received. Some
four years subse-
quently a home w as
opened on the Brandi Hill, for Leicester patients ; and at
the suggestion of the founders of this latter home the two in-
stitutions were amalgamated. The need for such an institu-
tion was so clearly proved by the large number of applica-
tions for admission, that in 1889 it was determined to
establish a home suitable for the district, and on a more
extended basis. We publish to-day the plans of the new
home, the founda-
tion-stone of which
was laid on August
3rd, 1893, by Her
Grace the Duchess of
Rutland.
The building is
planned very much
on the lines of a pri-
vate house, with
very little of the in-
stitution apparent.
This, for a house
such as this, which
is intended for the
use not only of pa-
tients recovering
from illness or opera-
tions, but also for
those in reed of rest
>OULLtRY\
BOOTS
knives] |
W'TCHEN
URINHLA
/foo/r '
OVEFf
Lflvzy
CHINR
\Lfivzy
S/TT/SVO?
/ROOM
O/N/N<3 H/RLL
\HHLL
\HfiLL\
WOMENS CORRIDOR.
SITTING
ROOM
FITTING
HOOM
SITTING
RtOOM
V EHANDAH
BED
ROOM
/BED
ROOM
BED
ROOM
/av'xav
3ED
ROOM
/svxiov
BED
ROOM
/8'0"X8O"
asDROOM
/a o"x iw
\BEDROOM
v,evxi+'o"
MfiTROt
/?> O "X /3 C
First Floor Plan.
CHARNWOOD FOREST CONVALESCENT
HOME.
feet above Ordnance
datum, and is four
acres in extent.
The home was
started in 1875 in a
cottage, where four
women were re-
ceived for a stay
of three weeks for
change of air and
rest. This modest
beginning proving
successful, a bed-
room in an adjoining
cottage was rented
in the following year
and two men patients
were received. Some
four years subse-
quently a home w as
home, the founda-
tion-stone of which
was laid on August
3rd, 1893, by Her
Grace the Duchess of
Rutland.
The building is
planned very much
on the lines of a pri-
vate house, with
very little of the in-
stitution apparent.
This, for a house
such as this, which
is intended for the
use not only of pa-
tients recovering
from illness or opera-
tions, but also for
those in reed of rest
Ground Floor Plan.
14 THE HOSPITAL. Oct. 6, 1894.
and change of air from overwork or other causes, is up
to a certain point the right line to work on. As we
have before pointed out, large wards or dormitories
are essentially out of place in a convalescent home ; there is
no economical need for them as there is in a hospital, and
there is every reason against them from the point of view of
discipline and the comfort of the patients.
The home is intended to provide for fifty patients, but
is furnished at present for only thirty. On the ground
floor is a large entrance-hall, designed to be used also
as a committee-room and a place wherein patients can
see their friends. On one side of this is the matron's
sitting-room, on the other a sitting-room, presumably for
the use of the nurses. The main corridor, which runs from
end to end of the building, is divided into three parts by two
sets of swing doors; one end being for women the other for
men, while the centre forms the common access to the dining
hall and the entrance. On the women's side is a sitting-room
with a bay window, and on the men's side two sitting-
rooms. The size of these rooms is very small for the number
of patients especially that for the women. The dining hall is
at the back and is common to both sexes. It is heated by hot
water coils. Built out at the back are the kitchen offices,
which are one storey only.
A verandah runs the whole length of the front, and is
accessible to all the patients' sitting-rooms. The earth
closets for the men, except one which is entered from the
lavatory, are outside the building ; but those for the women
are entered directly from their lavatory.
The first floor contains nine bed-rooms of varying sizes,
four bath-rooms, and two very small rooms off the half land-
ing of the stairs; and on the second floor are seven more
bed-rooms. With the exception of the bed-room marked
" Matron," we are not aware which of these rooms are de-
voted to patients and which to staff, but making the smallest
possible allowance for housing the necessary staff, the space
available for fifty patients must appear very inadequate, and
the dimensions of some of the rooms appear remarkably incon-
venient. The long corridor, too, lighted only at the ends, is
not an altogether desirable arrangement in a building where
some at least of the inmates must be more or less invalid.
In short, the plan appears to us to err on the side of being
too much of the private-house type. A little more light and
air in the corridor, and greater cubic space both in bed-rooms
and day-rooms, is much to be desired. Some provision in the
shape of earth closets ought surely to have been made on the
bed-room floor for night use, or for a patient confined to bed.
The cost of this home, including the purchase of the land
and the furnishing, was put at the astonishingly low
estimate of ?6,000. It would be interesting to know how
far this very remarkable estimate was verified by the result.
If the work was done for anything like this amount it reflects
much credit on the architect, Mr. George H. Barrowcliff, of
Loughborough.

				

## Figures and Tables

**Figure f1:**